# Perceptions of Child–Adult Relationship Enhancement (CARE) Training Usefulness for Educational, Behavioral, and Allied Health Professionals: Attitudes Toward Evidence-Based Practices

**DOI:** 10.1007/s10880-025-10093-1

**Published:** 2025-08-08

**Authors:** Julia L. Kiefer, Kristin J. Perry, Dustin E. Sarver, Emily-Anne S. del Rosario, Lauren B. Quetsch

**Affiliations:** 1 University of Arkansas at Fayetteville, Fayetteville, United States; 2 University of Oregon, Eugene, United States; 3 University of Mississippi Medical Center, Jackson, United States

**Keywords:** Child-Adult Relationship Enhancement, Behavioral health, Integrated care, Disruptive behaviors, Evidence-based practice

## Abstract

Child–Adult Relationship Enhancement (CARE) is an evidence-informed approach to promote positive child–adult relationships in youth with behavior problems or traumatic stress. Implementing CARE in community settings may extend accessibility to evidence-based practices (EBP) for children in underserved areas. The present study examined health professionals’ perceptions of CARE. Participants were 277 professionals from a statewide training initiative including early childhood educators (*n* = 178), allied health professionals (*n* = 48; speech, occupational, physical therapists), and behavioral health clinicians (*n* = 51) completing CARE training. Participants completed the Evidence-Based Practice Attitude Scale (Aarons (2004) Mental Health Services Research 6:61–74) (pre-training). Post-training, participants completed two scales created for this study which assessed participants perceptions of the training experience. Structural equation modeling evaluated differences in health professionals’ perceptions of CARE and EBP. CARE was the most well received by allied health professionals, who reported greater favorability of EBP relative to behavioral health clinicians (0.12, 95% CI [.04, .24]). Additionally, results indicated greater favorability of EBP-mediated perceived usefulness (0.17, 95% CI [.07, .31]). CARE is a well-received training for professionals working with youth. Interprofessional training may enhance developmental and behavioral outcomes for youth, and our findings suggest particular receptivity to CARE by allied health professionals and implicate EBP favorability as a key driver.

## Introduction

Emotional and disruptive behaviors are commonly observed in childhood, even among children with typical development (Cillessen & Lansu, 2014; Evans et al., 2019; Sheppard et al., 2016; Thornberg, 2013). Left unaddressed, more significant behaviors (e.g., traumatic stress, extreme aggression) have been linked with problematic trajectories such as the development of conduct problems (McCart & Sheidow, 2016). In the last two decades, a shift toward creating and strengthening universal mental health programs has occurred. These programs have often been embedded within a stepped-care public health model aimed at addressing youth mental health across the prevention, promotion, and intervention continuum (Cross & Hickie, 2017). Such an approach is vital given the high rates of adverse childhood experiences among youth (Finkelhor, 2020) and lack of mental health care access available in the U.S (Whitney & Peterson, 2019; Costello et al., 2014). Universal approaches allow professionals to apply the same preventative technique or interventions to all youth in community settings (e.g., school, clinics).

One such universal prevention approach for youth is Child–Adult Relationship Enhancement (CARE). CARE is an evidence-informed training program that increases adults’ effectiveness in their interactions with all youth aged 2–18 years, including those who may be exhibiting disruptive behaviors or with trauma experiences (Gurwitch et al., 2016; National Child Traumatic Stress Network, 2020). Backed by the same theories and strategies used in evidence-based parent-training programs including Parent–Child Interaction Therapy (Funderburk & Eyberg, 2011) and The Incredible Years (Webster-Stratton, 2001), CARE seeks to promote youth’s positive behavior, attachment, and relationships with important figures in their lives (e.g., caregivers, teachers, health providers). The specific techniques taught in CARE have over 50 years of evidence, and recent evidence of their packaging in the CARE training format indicates CARE has potential to reduce child maltreatment and children’s disruptive behaviors (Schilling et al., 2017, 2024; Wood et al., 2021). Importantly, CARE can be implemented in a variety of settings such as daycares, schools, outpatient clinics, or child welfare agencies to enhance its reach and impact as a universal program (Gurwitch et al., 2016; NCTSN, 2020).

To successfully implement CARE in these settings, CARE trainings are disseminated to professionals through workshop-style trainings. Given evidence that workshops alone generally increase knowledge but not behavioral changes (Fabiano et al., 2013), CARE trainings also include well-established adult learning procedures such as role play, rehearsal feedback, and live skills coaching, which ensure greater likelihood for trainee skills acquisition (Gurwitch et al., 2016). During CARE trainings, participants learn skills called “the P’s and Q’s,”—a simplified version of the “PRIDE” skills taught in Parent–Child Interaction Therapy (Gurwitch et al., 2016). The 3 “Q” skills taught address harsh/coercive interactions and include (1) Quash the need to lead, (2) Quit unnecessary questions, and (3) Quiet the criticisms. The 3 “P” skills taught address warmth and fostering positive relationships and include: (1) Praise the child for good behavior, (2) Paraphrase what the child is telling you, and (3) Point out the child’s positive actions. Participants are also taught strategic ignoring skills and other non-coercive discipline techniques in the face of non-compliance (Gurwitch et al., 2016).

In recent years, there has been a dramatic shift toward integrated healthcare services in education, clinic, and community settings, which underscores the importance of interprofessional education and training provided by psychologists to other healthcare disciplines, including universal approaches like CARE (Cubic et al., 2012; McDaniel et al., 2014). Though previous studies have focused training on specific populations such as medical professionals or trainees (Murphy et al., 2019; Scott et al., 2024), researchers have hypothesized CARE to be effective for use by professionals from any setting and with youth from a broad age range (Gurwitch et al., 2016). Additionally, although CARE’s relatively short (single day) training model allows for easy dissemination relative to other evidence-based practices (EBP; Blair et al., 2019; Onken et al., 2013; Webb et al., 2016), little evidence exists that examines perceived usefulness of CARE from the perspectives of different professional backgrounds (National Child Traumatic Stress Network, 2020). Additionally, though CARE skills may be implemented by healthcare professionals, much of the previous research examines CARE from the perspective of caregivers (e.g., Schilling et al., 2017, 2024; Wood et al., 2021). Though generally well received by caregivers (Schilling et al., 2017), examination of how healthcare professionals perceive and engage with CARE is warranted.

Research on other intervention models implemented in similar settings suggests that individual attitudes of EBP are a key factor impacting successful delivery and use of EBP (Locke et al., 2019; Lyon & Bruns, 2019). Indeed, training in and penetrance of EBP vary across disciplines (Marlowe et al., 2020; Snibsøer et al., 2018). As such, understanding attitudes toward EBP may be helpful in explaining differences in perceptions regarding CARE usefulness and potentially informing its implementation within various professional communities (Eslinger et al., 2020). Thus, the current study had two aims: (1) to examine perceptions of CARE trainings and its usefulness within the professional settings for three diverse disciplines to which positive behavioral support approaches are important (early childhood educators, allied health providers, and behavioral health clinicians); and (2) determine whether differences in perceptions of training usefulness are mediated by professionals’ attitudes toward EBP.

## Methods

### Procedure

The current study was drawn from a larger retrospective investigation concerning statewide implementation of CARE in Mississippi to diverse educational, health, and behavioral health providers. The larger retrospective study contained CARE training participants from other areas (e.g., nurses, foster care parents, psychiatric inpatient technicians) or contained support staff professionals within the settings included here (e.g., school bus drivers, receptionists). The specific professional areas were selected by convenience for the study based on being the primary training targets of the larger overall project and due to their similarities in providing direct services to children. Trainings were conducted as single day professional development workshops within the trainees’ facility. Trainings included small group discussions, video demonstrations, and opportunities to practice skills with live coaching from a CARE trainer as well as 1–3 subsequent coaching sessions. Trainings were conducted by licensed clinicians within the University of Mississippi Medical Center trained to facilitate CARE. Trainees completed measures pre-training and immediately post-training the same day. Procedures were IRB approved (UMMC-IRB-2019-0272), and data and analytic syntax are publicly available for peer review (https://osf.io/exzku/?view_only=eb71980491be492caf3cd986f2ffee85).

### Participants

Participants for the current study (*N* = 274) were taken from the CARE trainings that occurred with three different professional areas: (1) early childhood educators (*n* = 176); (2) allied health providers (speech, occupational, and physical therapists; *n* = 47); and (3) masters-level behavioral health counselors (*n* = 51). Trainings were both required and elective for participants depending on their profession. Specifically, early child educators and behavioral health providers were primarily required by their institution to participate in trainings, while allied health providers were primarily elective. Participants were primarily female (97.1%) and African American/Black (62.4%), and 19.3% had a GED or high school diploma (Table 1). Professional disciplines for the sample are given in Table 1.

### Measures

Prior to the CARE trainings, the 15-item Evidence-Based Practice Attitude Scale (EBPAS; Aarons, 2004) was used to assess EBP attitudes. The EBPAS quantifies clinicians’ attitudes toward using treatment manuals, empirically supported interventions, and evidence-based interventions through a 5-point scale (0 = *not at all* to 4 = *a very great extent*). The measure produces four subscales: Requirements (professional use of a new intervention if it was an agency or state requirement), Appeal (professional use of a new treatment if it makes intuitive sense or if colleagues approve of it), Openness (willingness to use new treatment types), and Divergence (professional perception of evidence-based interventions as neither important nor useful). Scores on the EBPAS are derived from participant responses to items, with higher scores indicating higher agreement with items in each area (i.e., Requirements, Appeal, Openness, Divergence). Minor item wording modifications were made to ensure fit to professional setting (i.e., educator/student; clinician/patient). In prior research, the EBPAS demonstrated acceptable ecological validity, content validity, and factor structure (Aarons, 2004; Aarons et al., 2007, 2010).

Post-training, participants responded to two scales assessing how useful they found the training experience. A six-item overall training usefulness scale assessed participants’ evaluation of training overall (e.g., ‘This training increased my skills in this area’). A 7-item specific skill usefulness scale assessed participants evaluation of specific CARE training skills (e.g., ‘I have learned new approaches to using praise’). Post-training scales were created by clinical researchers originally involved in the development of CARE to assess post-training usefulness of the program consistent with continuing education evaluation. Scores were totaled across the measures with higher scores reflecting more favorable evaluations of CARE’s usefulness. Both are included as supplementary materials.

All scales were reliable and demonstrated acceptable internal consistency (Cronbach’s α >.78).

### Analytic Strategy

Descriptive statistics were examined across professional groups (Table 1). Outliers were assessed using skew and kurtosis variables and were adjusted to ± 3 standard deviations from the mean (Klein, 2016).

To examine the first aim of the study, a four-factor measurement model was tested for the EBPAS, congruent with previous research (Aarons et al., 2010). Difference coding was conducted to test comparisons between occupations. Two variables were created. Variable 1 tested comparisons between early education educators [code = 2] and other professionals [i.e., allied health/counselors = − 1]. Variable 2 tested comparisons between allied relative to behavioral health professionals [allied health = 1, behavioral health = − 1, early education educators = 0]. Next, a path analysis model, which tested the mediating effect of EBP between professional group and post-training CARE perceptions, was conducted with maximum likelihood estimation in Mplus 8.7 (e.g., Muthén & Muthén, 1998–2022). To test model fit, the likelihood ratio *χ*^2^ was used in addition with the fit indices, standardized root mean square residual (SRMR), comparative fit index (CFI), and the root mean squared error of approximation (RMSEA). SRMR values less than .08, CFI values greater than .90, and RMSEA values less than .08 indicated acceptable model fit (Hu & Bentler, 1999). Indirect effects were tested with bias-corrected 95% confidence intervals using 5000 bootstrapped samples. First, a confirmatory factor analysis was used to examine the EBPAS factors. Next, a path model was tested using an overall EBP attitudes factor. Finally, a path model was examined using the EBP attitude subscale factors.

## Results

**Fig. 1:**
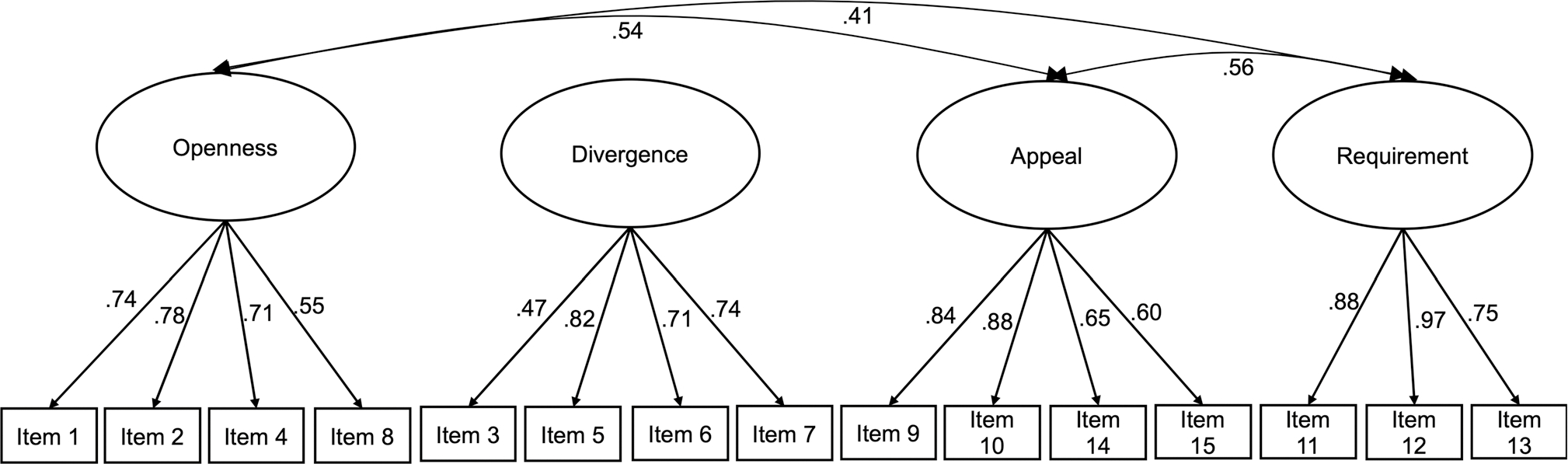
Four-factor measurements model *Note*. Items and subscales are based on Aarons (2004).

The measurement model with the four EBPAS attitude factors provided a good fit to the data [*χ*^2^ (84) = 143.54, *p* <.001, CFI = 0.96, SRMR =.05, RMSEA =.05]. All factor loadings were substantial and significant (.55–.88). Given that divergence was not associated with other factors (*rs* =.00-.09; *ps*.25–1.0), a higher order EBP favorability factor was created of the three other subscales, which provided an acceptable fit to the data [*χ*^2^ (86) = 147.43, *p* <.001, CFI = 0.96, SRMR =.06, RMSEA =.05;; See Fig. 1].

**Fig. 2:**
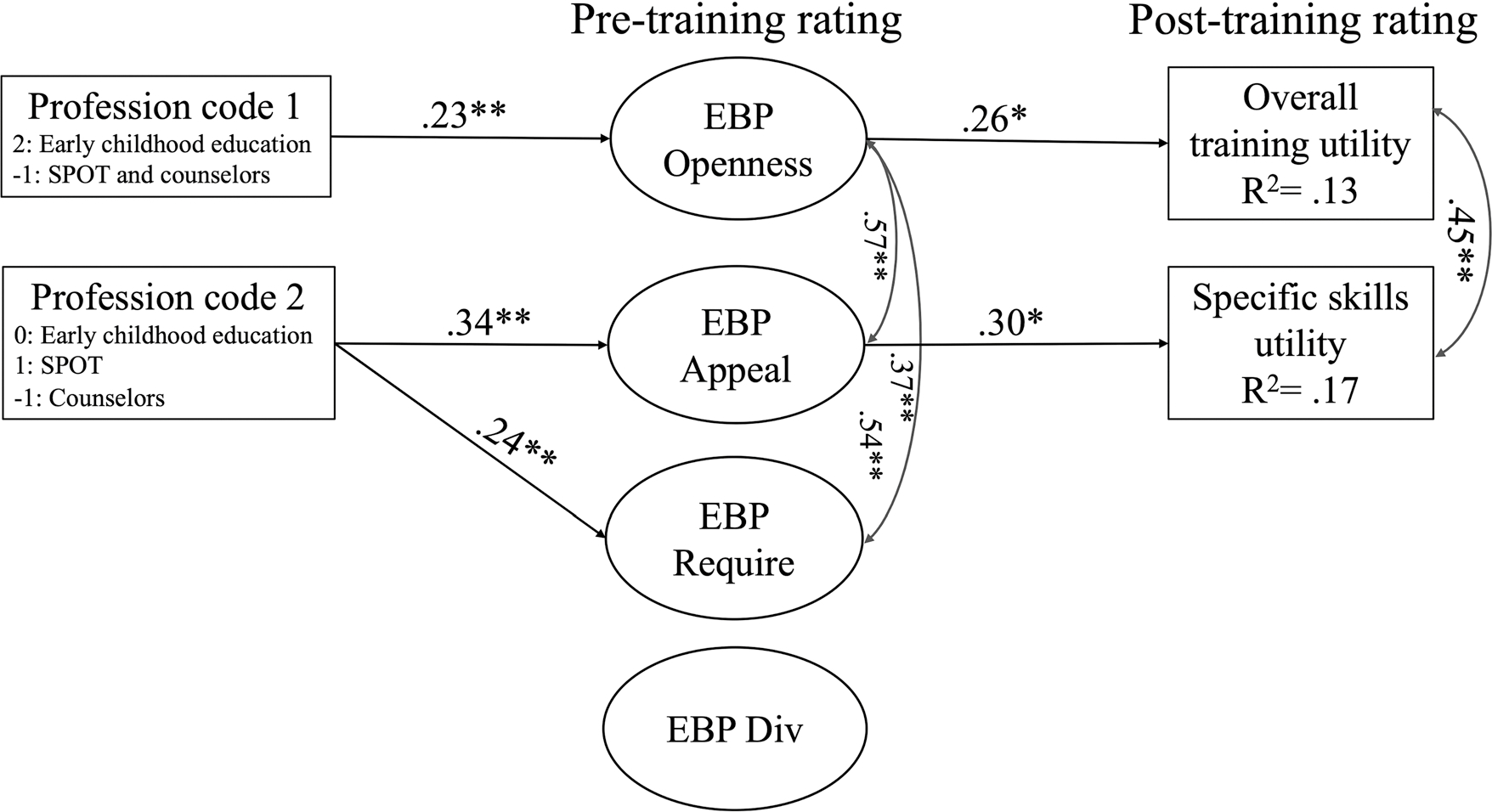
Indirect effects structural model *Notes*. *p < .05, **p < .01. All models controlled for education (dummy coded) and years of experience. SPOT = Speech, occupational, or physical therapist or assistant (Allied Health Providers).

A structural model with the EBP favorability higher order factor provided an acceptable fit to the data [*χ*^2^ (139) = 247.86, *p* <.001, CFI = 0.94, SRMR =.06, RMSEA =.05; see Fig. 2 for standardized estimates]. Allied health professionals reported greater favorability of evidence-based practices than behavioral health clinicians, which in turn was associated with higher post-training ratings in the usefulness of the training overall (0.12, 95% CI [.04,.24]) and of specific skills (0.17, 95% CI [.07,.31]).

The structural model with the EBPAS subscale factors also provided an acceptable fit to the data [*χ*^2^ (129) = 208.37, *p* <.001, CFI = 0.96, SRMR =.05, RMSEA =.05]. The same paths for divergence emerged as significant and are therefore not shown in the figure. Allied health professionals reported more appeal to EBP prior to receiving CARE training relative to behavioral health professionals, which in turn was associated with higher post-training ratings in utility of specific skills (0.14, 95% CI [.04,.32]). Second, there was evidence of competitive mediation (Zhao et al., 2010) for the association between the first contrast variable (i.e., comparisons between early education educators and other professionals) and post-training ratings in overall training utility. The direct effect indicates that early childhood educators had lower post-training ratings in the utility of the training overall relative to the other professions, whereas the indirect effect (0.04, 95% CI [.01,.09]) suggests that early childhood educators reported more openness to EBPs, which was associated with higher ratings in the training overall.

## Discussion

Based on evidence-based parent-training interventions, CARE is a relatively new universal prevention approach aimed at promoting positive interaction skills for adults responsible for assisting any youth, though CARE may be particularly advantageous to youth who exhibit disruptive behaviors or traumatic stress. CARE offers a universal training approach in behavior management skills, which may be beneficial for health professionals who may encounter youth with disruptive behaviors but may have not otherwise had training in EBP. Indeed, CARE is easily accessible compared to other programs which require extensive training and consultation (Gurwitch et al., 2016) and have particular utility as an easily disseminated prevention strategy to curb the development of more severe behavior and psychological problems both universally and among more at-risk populations (e.g., youth requiring developmental supports). Its accessibility also has promise given increased disparities in access to mental health programs and practices for youth from rural and underserved areas (Elkin et al., 2018). With integrated behavioral healthcare gaining traction in service delivery (Asarnow et al., 2015), CARE may be especially poised to meet the recognized need of embedding behavioral healthcare principles and strategies in interprofessional training experiences (Cubic et al., 2012; McDaniel et al., 2014). As such, CARE has the potential to enhance integrated healthcare systems by improving the quality of services delivered across various professions serving children. However, CARE will offer little utility if providers are unwilling to implement the program or find it useful. Consequently, the current study documented differences in the perceptions of general training usefulness and of specific CARE skills between providers from three different professions where CARE may be particularly relevant (i.e., early childhood educators, allied health, and behavioral health).

Consistent with previous research (Gurwitch et al., 2016; Schilling et al., 2017, 2024; Wood et al., 2021), results from the current study indicated that allied health professionals (speech, physical, and occupational therapists) found the CARE training to be most useful. Indeed, while CARE was highly rate across all groups, allied health professionals reported higher overall utility of CARE training than behavioral health professionals, which was mediated by the appeal of EBP. Additionally, indirect effects of the mediation in the structural model (Fig. 2) indicated that allied health professionals reported higher favorability of overall EBPs than behavioral health or early childhood educators. In turn, overall EBP favorability was linked to higher post-training ratings of the usefulness of both overall and specific skills, and usefulness of specific skills appeared to be driven singularly by perceived appeal of EBPs. However, although early childhood educators perceived overall usefulness of CARE to be lower than allied health professionals, early childhood educators also reported greater openness to EBPs, which was associated with higher usefulness ratings. This mediation effect was weak relative to the prior meditation effect and only emerged in the second path model. Therefore, the evidence for competitive mediation indicates the effects be interpreted with caution. Optimistically, this would suggest that enhancing the connection between CARE skills and EBPs during training may be especially helpful for early childhood educators.

CARE is not a manualized treatment, but instead a set of skills providers can use with all youth, including those exhibiting disruptive or trauma-based behaviors (Gurwitch et al., 2016). As such, allied health professionals may have found CARE to be appealing and easy to implement within their practice given they often receive less extensive training in mental health or behavioral management practices (Baier et al., 2018) despite seeing elevated rates of behavioral difficulties (Simó-Pinatella et al., 2017). For instance, the inclusion of interpersonal interaction, paraphrasing, and elaboration as CARE skills are common core elements of speech-language interventions (Dwight, 2022). Moreover, previous research has found that some professionals, specifically occupational therapists, worry about balancing their clinical activities while staying within the scope of their competence when working with individuals with mental health needs (Baier et al., 2018). As such, CARE may be especially useful in allowing allied health professionals to still practice within the bounds of their competence, while also helping support the needs of youth with behavioral and emotional difficulties.

Findings indicated that professionals from all disciplines found CARE to be highly useful, but allied health professionals found CARE especially useful. However, results indicated that early childhood educators expressed lower utility of CARE training. As such, results of the current study suggest that to the extent early childhood educators are open to EBP, their perceptions of usefulness of EBP increased. Lower utility of CARE training reported by early childhood educators may stem from the availability and favorability of other, more established EBP specifically designed for classroom behavior managements, such as Positive Behavioral Interventions and Supports (PBIS; Palmer & Noltemeyer, 2019; Sugai & Horner, 2002), which is designed to increase youth’s prosocial behaviors, academic performance, and overall school climate. Whereas early childhood educators likely have increased accessibility of other EBP such as PBIS, allied health professionals often do not, which may explain the significant differences of perceived usefulness of CARE found between groups. Alternatively, this finding may reflect the range and overall lower educational attainment by early childhood educators in this study (i.e., 61% non-college graduates), wherein exposure to EBP principles may be lower. Thus, the greater the association with EBP favorability and usefulness may indicate CARE as a better fit for those with more EBP exposure.

Combined with lower openness to EBP among behavioral and allied health providers, and lower EBP appeal among educators, CARE trainings could specifically benefit from incorporating strategies within training that target and increase EBP openness and appeal. Indeed, CARE training itself may benefit by addressing providers attitudes toward EBP prior to, or in the early stages of training efforts. Additionally, future training efforts for CARE may consider offering dedicated space during training days for practice in implementation after training, and follow-up with providers after implementation efforts are underway in their practices. Future training efforts should also continue to assess providers acceptance of EBP with the goal to bolster EBP attitudes and enhance uptake, as other studies have previously reported less favorable attitudes to EBP adoption among mental health professionals compared to education-based providers (Stahmer & Aarons, 2009). Future research may also explore the efficacy of incorporating CARE as a training experience for early childhood educators or allied health professionals as a part of their educational training, rather than an additional training post-graduation when EBP attitudes are likely more formative.

This study had several strengths including its novel focus on understanding perspectives of individuals from diverse professional identities including speech, physical, and occupational therapists, as well as early childhood educators and behavioral health clinicians. The study’s inclusion of a demographically and educationally diverse sample is a strength as well. Despite these strengths, several important limitations warrant consideration. First, the outcomes from this study offered perspectives from practitioners who were primarily female, with very few male participants (less than 10%); however, while this sample may be largely female identifying, it also largely represents the demographic of each professions’ typical background based on U.S. provider statistics (U.S. Department of Health and Human Services, 2017). Additionally, the current study did not examine whether providers implemented CARE in their practices after training. Although providers may have found the training to be useful, previous research indicates that providers from interdisciplinary behavioral health teams (e.g., occupational, speech therapists) may experience barriers to implementing evidence-based treatments, such as feeling a lack of knowledge or supervision in implementation (Nelson & Steele, 2007). As such, future research should also longitudinally examine the outcomes of CARE training, such as examining whether professionals continue to use aspects of CARE training in their own practices. A prior presentation among a small subset of the allied health providers encouragingly did demonstrate improved behaviors after implementation (Sarver et al., 2019). Researchers should also examine more closely which aspects of CARE training seem to be most useful for professionals and barriers to implementing CARE within their own practice. Additionally, results from the competitive mediation analyses suggested a high likelihood of an omitted mediator in the model (Zhao et al., 2010). As such, future research should examine other potential mediators to explain differences between early childhood educators and their perception of CARE training usefulness.

Overall, CARE is an evidence-informed prevention approach well situated for inclusion among stepped-care public health models focused on universal mental health promotion and prevention across the care continuum (Cross & Hickie, 2017; Gurwitch et al., 2016). CARE is unique in that it offers a universal technique for providers from different disciplines to enhance direct care in interactions with youth who may not otherwise come in contact with EBP. CARE was particularly well received by allied health professionals, suggesting future training efforts should focus on increased dissemination to other health disciplines to ensure increased access to behavioral health care for youth. Effectively disseminating universal approaches to meet the needs of as many youths as possible will require multidisciplinary teams of professionals who interact with youth across contexts (Lee et al., 2016). As such, broad dissemination efforts for CARE in the form of interprofessional training to other disciplines may enhance alignment among integrated care professions. In turn, use of CARE skills may promote greater engagement in developmental interventions that may translate to better developmental and behavioral health outcomes for youth. Future research should continue to examine the effectiveness of CARE and its immediate and long-term efficacy in different contexts.

## Supplementary Material

Supplementary Material

**Supplementary Information** The online version contains supplementary material available at https://doi.org/10.1007/s10880-025-10093-1.

## Figures and Tables

**Table 1:** Group differences in demographic and primary variables

	Allied health professional *n* = 47		Early childhood educator *n* = 176		Counselor *n* = 51					

Demographic variables	*M* or %	*SD*	*M* or %	*SD*	*M* or %	*SD*	*F*/χ^2^	Adjusted R^2^	Missing Data %	Groups differed

Years of experience	10.43	9.72	10.32	9.37	8.01	6.99	1.35	.003		
Race/ethnicity	10.87% Black		71.59% Black		80% Black		**68.15** **			Allied health vs. Educator & Counselor
Education	0.00% GED/Diploma 6.38% AA/cert 55.32% Bachelors 38.30% Graduate		30.11% GED/Diploma 31.25% AA/Cert 26.13% Bachelors 12.50% Graduate		0.00% GED/Diploma 0.00% AA/cert 37.25% Bachelors 62.75% Graduate		**104.54** **			Allied health vs. Educator vs. Counselor
Professional Discipline	40.43% Occupational Therapist 36.17% Speech Pathologist 23.40% Physical Therapist		81.25% Teacher 18.75% Teaching Assistant							

Primary variables										

Overall training utility	3.86	0.23	3.72	0.40	3.74	0.40	**2.64**†	.01	3.28%	Allied health vs. educator
Specific skills training utility	3.68	0.32	3.53	0.47	3.60	0.43	2.32	.01	4.01%	
EBPAS openness	2.99	0.71	3.22	0.63	2.98	0.59	**4.08** *	.02	1.09%	Educator vs. Allied health & Counselor
EBPAS appeal	3.46	0.56	3.00	0.64	2.77	0.95	**12.42** **	.08	7.47%	Allied health vs. Educator vs. Counselor
EBPAS divergence	3.08	0.71	2.43	0.99	2.86	0.89	**10.96** **	.07	1.46%	Allied health & Counselor vs. Educator
EBPAS requirements	3.34	0.85	3.23	0.81	2.86	1.05	**4.21** *	.02	5.11%	Allied health & Teacher vs. Educator

Note.

* *p* < .05,

** *p* < .01. AA = Associate’s degree, Cert = Professional certification, Allied health professional = Speech, occupational, or physical therapist or assistant. Bold values indicate a significant group difference.

## Data Availability

Data and analytic syntax are publicly available for peer review at https://osf.io/exzku/?view_only=eb71980491be492caf3cd986f2ffee85.
