# Supplementary material for: Perceptions of Child–Adult Relationship Enhancement (CARE) Training Usefulness for Educational, Behavioral, and Allied Health Professionals: Attitudes Toward Evidence-Based Practices
Source: J Clin Psychol Med Settings. Author manuscript; Available in PMC 2026 Jan 27. (PMC12638389; doi:10.1007/s10880-025-10093-1)
Supplement: Supplementary Material [file NIHMS2116740-supplement-Supplementary_Material.docx]

**Child Adult Relationship Enhancement (CARE) Post-Evaluation**

**Please rate this workshop by marking one answer in each category.**

|  | **Did Not Satisfy Expectations** | **Somewhat Satisfied Expectations** | **Mostly Satisfied Expectations** | **Completely Satisfied Expectations** |
| --- | --- | --- | --- | --- |
| 1. This training increased my knowledge of this subject. |  |  |  |  |
| 1. This training increased my skills in this area. |  |  |  |  |
| 1. Stated goals and learning objectives were met. |  |  |  |  |
| 1. The information was delivered in an organized and understandable manner. |  |  |  |  |
| 1. The presenter was knowledgeable in this subject. |  |  |  |  |
| 1. Educational aids contributed to overall learning. |  |  |  |  |

1. Any suggestions for future trainings/presenters:

___________________________________________________________________________________________________________________________________________________________________________________________________________________________________________________

1. Any additional comments:

___________________________________________________________________________________________________________________________________________________________________________________________________________________________________________________

**Child Adult Relationship Enhancement (CARE) Post-Evaluation**

**Please rate this training by marking one answer in each category.**

|  | **Strongly Disagree** | **Disagree** | **Agree** | **Strongly Agree** |
| --- | --- | --- | --- | --- |
| 1. The **3P’s skills** can be useful with a child. |  |  |  |  |
| 1. The **Avoid skills** can be useful with a child. |  |  |  |  |
| 1. The **Ignore skills** can be useful with a child. |  |  |  |  |
| 1. I have learned **new approaches** to using praise (i.e., making praises labeled, praising behaviors I want to see more of) that I have not previously used. |  |  |  |  |
| 1. I have learned **new approaches** to describing a child’s behavior (i.e., pointing out what the child is doing by stating, “You are…”) that I have not previously used. |  |  |  |  |
| 1. I have learned **new approaches** to paraphrasing a child’s appropriate talk (i.e., paraphrasing to enhance language skills, avoiding making reflections a question) that I have not previously used. |  |  |  |  |
| 1. I feel comfortable implementing the CARE skills on my own. |  |  |  |  |

1. Any additional comments:

___________________________________________________________________________________________________________________________________________________________________________________________________________________________________________________
