# Supplementary material for: Perceptions of Child–Adult Relationship Enhancement (CARE) Training Usefulness for Educational, Behavioral, and Allied Health Professionals: Attitudes Toward Evidence-Based Practices
Source: J Clin Psychol Med Settings. 2025 Aug 8;32(4):663–71. doi: 10.1007/s10880-025-10093-1 (PMC12638389; doi:10.1007/s10880-025-10093-1)
Supplement: Supplementary file 1 [file 10880_2025_10093_MOESM1_ESM.docx]

**Child Adult Relationship Enhancement (CARE) Post-Evaluation**

**Please rate this workshop by marking one answer in each category.** 

|  | **Did Not Satisfy Expectations** | **Somewhat Satisfied Expectations** | **Mostly Satisfied Expectations** | **Completely Satisfied Expectations** |
| --- | --- | --- | --- | --- |
| 1. This training increased my knowledge of this subject. |  |  |  |  |
| 2. This training increased my skills in this area. |  |  |  |  |
| 3. Stated goals and learning objectives were met. |  |  |  |  |
| 4. The information was delivered in an organized and understandable manner. |  |  |  |  |
| 5. The presenter was knowledgeable in this subject. |  |  |  |  |
| 6. Educational aids contributed to overall learning. |  |  |  |  |

7. Any suggestions for future trainings/presenters:

___________________________________________________________________________________________________________________________________________________________________________________________________________________________________________________

8. Any additional comments:

___________________________________________________________________________________________________________________________________________________________________________________________________________________________________________________

**Child Adult Relationship Enhancement (CARE) Post-Evaluation**

**Please rate this training by marking one answer in each category.**

|  | **Strongly Disagree** | **Disagree** | **Agree** | **Strongly Agree** |
| --- | --- | --- | --- | --- |
| 9. The **3P’s skills** can be useful with a child. |  |  |  |  |
| 10. The **Avoid skills** can be useful with a child. |  |  |  |  |
| 11. The **Ignore skills** can be useful with a child. |  |  |  |  |
| 12. I have learned **new approaches** to using praise (i.e., making praises labeled, praising behaviors I want to see more of) that I have not previously used. |  |  |  |  |
| 13. I have learned **new approaches** to describing a child’s behavior (i.e., pointing out what the child is doing by stating, “You are…”) that I have not previously used. |  |  |  |  |
| 14. I have learned **new approaches** to paraphrasing a child’s appropriate talk (i.e., paraphrasing to enhance language skills, avoiding making reflections a question) that I have not previously used. |  |  |  |  |
| 15. I feel comfortable implementing the CARE skills on my own. |  |  |  |  |

	16. Any additional comments:

___________________________________________________________________________________________________________________________________________________________________________________________________________________________________________________
